# Vortex-actuated pre-enrichment accelerates human regulatory T cell sorting and improves early fitness

**DOI:** 10.1016/j.omta.2026.201667

**Published:** 2026-01-09

**Authors:** Quan Yao Ho, Hisashi Hashimoto, Joanna Hester, Fadi Issa

**Affiliations:** 1 Translational Research Immunology Group, Nuffield Department of Surgical Sciences, University of Oxford, Oxford OX3 9DU, UK; 2 Department of Renal Medicine, Singapore General Hospital, Singapore 169608, Singapore; 3 Chinese Academy of Medical Sciences Oxford Institute, University of Oxford, Oxford OX3 7BN, UK

**Keywords:** cell separation, T-Lymphocytes, regulatory, cell- and Tissue-based therapy, magnetic-activated Cell sorting, vortex-actuated cell sorting, fluorescence-activated cell sorting, cell Survival

## Abstract

Cell selection is critical for regulatory T cell (Treg) therapy manufacturing. Pre-enrichment can reduce sorting time, yet data comparing different methods remain scarce. Highway1, a Good Manufacturing Practice (GMP)-compliant cell sorter, separates cells using transient vortices (i.e., vortex-actuated cell sorting [VACS]), which avoids exposure to magnetic nanoparticles and may improve cell viability compared to immunomagnetic separation (IMS). We compared VACS with IMS enrichment before sorting for human Tregs. Tregs were sorted from peripheral blood mononuclear cells isolated from donor leukocyte cones directly using the Highway1 (DP), CD4+CD25+ VACS enrichment then VACS (VP), or CD25+ IMS positive selection then VACS (25MP), and expanded with anti-CD3+/CD28+ stimulation beads, IL-2, and rapamycin. VP, 25MP, and DP produced Tregs of comparable purity. VP reduced sorting time but decreased yield compared to DP. VP resulted in fewer late apoptotic and hypoproliferative, IL-2 hyporesponsive cells, and generated Tregs that expanded more than 25MP. Phenotypic markers and suppression function did not differ after 21 days of expansion. CD4+CD25+ VACS enrichment reduced the time needed for Treg sorting compared to direct VACS and generated Tregs that expanded more than those enriched by CD25+ IMS selection, supporting adoption of a single-platform, closed VACS workflow and justifying validation at GMP scale.

## Graphical abstract

**Figure:**
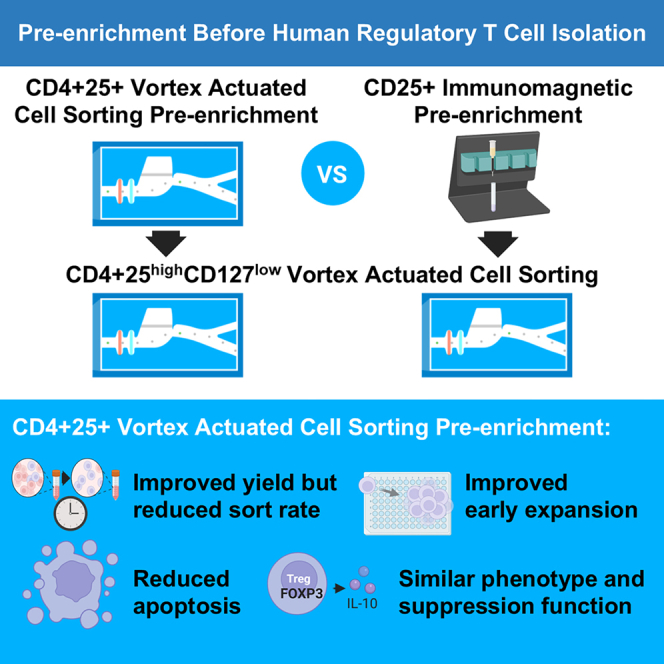

## Teaser

Ho and colleagues described a pre-enrichment method for human regulatory T cell isolation using a microfluidics-based cell sorter that sorts cells using transient vortices. Compared to conventional CD25+ immunomagnetic pre-enrichment, this method improved cell yield and generated regulatory T cells that expanded more robustly.

## Introduction

Regulatory T cells (Tregs) are a subset of naturally occurring CD4+ T cells that suppress excessive immune responses and have demonstrated promising results for a variety of indications, including the prevention of transplant rejection and treatment of autoimmune diseases.^1^^,^^2^^,^^3^^,^^4^ As Tregs are relatively rare and have reduced viability *ex vivo*, high-quality cell selection is crucial to produce Tregs of sufficient quantity, purity, and viability for both research and clinical applications.^5^

Tregs, including Treg cell products used in current clinical trials, can be isolated using immunomagnetic separation (IMS).^6^^,^^7^^,^^8^^,^^9^^,^^10^^,^^11^ CD4+CD25^high^ cells are commonly enriched from peripheral blood mononuclear cells (PBMCs) isolated from patients’ blood by first removing unwanted cells (e.g., CD8+ T cells and CD19+ B cells), then positively selecting for CD25+ cells using magnetic fields after tagging cells to superparamagnetic iron oxide nanoparticles conjugated to specific antibodies. However, there are concerns about the viability of Tregs isolated by IMS.^12^^,^^13^^,^^14^^,^^15^^,^^16^ A higher degree of purity may also be needed, especially in gene-edited antigen-specific Treg therapies, to reduce the risk of adverse effects due to contamination by pro-inflammatory conventional T cells.^17^^,^^18^

Tregs may alternatively be sorted using fluorescence-activated cell sorting (FACS).^19^^,^^20^^,^^21^^,^^22^ In conventional FACS sorters, cells are hydrodynamically focused, then separated into discrete droplets by passing the fluid stream through a vibrating nozzle. The droplets are electrostatically charged and separated by electrical fields based on signals from surface markers marked by fluorescent antibodies. To ensure sterility and meet strict Good Manufacturing Practice (GMP) requirements, the new generation of FACS sorters typically features single-use, fully closed microfluidic circuits, where cells are separated by diverting the fluid stream using technologies such as an alternating “trapdoor” mechanism or through aspiration by generating negative pressures.^22^^,^^23^^,^^24^ However, unlike IMS, FACS is unable to process large numbers of cells in bulk. Therefore, clinical-scale Treg FACS is slow and exposes cells to conditions that may impair viability and expansion.^12^^,^^25^ Pre-enrichment helps with speed but may compromise viability when using CD25+ IMS.^22^^,^^26^^,^^27^ Moreover, cell sorting using different methods and devices can be cumbersome and expensive.^12^^,^^16^ Importantly, data on alternative enrichment methods and those comparing different sorting techniques for Tregs are limited.

Highway1 is a fully closed, GMP-compliant, microfluidics-based cell sorter that identifies target cells via conventional flow cytometry, then separates them using transient vortices (i.e., vortex-actuated cell sorting [VACS]). Cells sorted using VACS avoid exposure to magnetic nanoparticles and thus may have improved viability compared to those sorted by IMS.^28^^,^^29^ In “enrichment” mode, cells are processed at a higher rate at lower purity by sorting target cells together with adjacent non-target cells. In contrast, the sorting rate in “purity” mode is reduced but at a higher degree of purity, since only target cells are sorted.

Currently, numerous Treg isolation protocols utilize CD25+ IMS enrichment prior to FACS. VACS enrichment prior to VACS sorting may be an attractive alternative, since it will simplify the sorting workflow, avoid unwanted effects due to exposure to magnetic nanoparticles, and keep sorting time manageable. We hypothesized that enriching for CD4+CD25+ cells using VACS “enrichment mode” before VACS can produce Tregs with purity and function that are comparable to those produced by CD25+ IMS enrichment. As such, we conducted a series of experiments to compare the performance of VACS and IMS enrichment before VACS of human Tregs.

## Results

### CD4+CD25+ VACS enrichment for human Tregs produces Tregs with comparable purity and increased yield but with lower sort rate compared to CD25+ IMS enrichment

**Figure 1:**
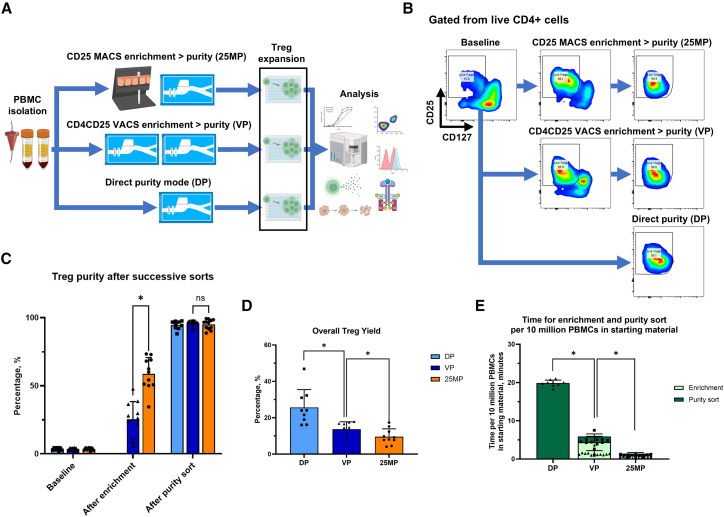
Sorting performance comparing CD4+CD25+ vortex-actuated cell sorting enrichment followed by vortex-actuated cell sorting purity sort, CD25+ immunomagnetic separation enrichment followed by vortex-actuated cell sorting purity sort, or direct vortex-actuated cell sorting purity sort for human regulatory T cells (A) Overview of experimental design. (B) Representative flow cytometric plots after regulatory T cell (Treg) enrichment and/or purity sorts, with percentages of CD4+CD25^high^CD127^low^ of live CD4+ cells. (C) Treg purity, defined as the percentage of CD4+CD25^high^CD127^low^ cells of total live cells, following successive enrichment and purity sorts. (D) Overall Treg yield is defined as the percentage of Tregs recovered in the final product relative to the number of Tregs in the PBMC starting material. (E) Time required for enrichment and purity sort per 10 million PBMCs in the starting material. Direct VACS purity sort (DP), CD4+CD25+ vortex-actuated cell sorting enrichment followed by VACS purity sort (VP), or CD25+ immunomagnetic separation (IMS) enrichment followed by VACS purity sort (25MP). Each symbol represent individual data points; bars indicate the mean ± SD. For (C)–(E) paired *t* tests were performed. “∗” denotes a significance of *p* < 0.05, *n* = 12 donors

To compare the performance between VACS and IMS enrichment (Figures 1A and 1B), we sorted 80–400 × 10^6^ PBMCs freshly isolated from donor leukocyte cones with either CD4+CD25+ VACS in enrichment mode (VP) or CD25+ IMS positive selection (25MP), before immediately sorting for Tregs using VACS in purity mode. For comparison, we also sorted 40 × 10^6^ PBMCs directly using VACS purity mode (DP) in the same setting (Table S1).

The different enrichment modalities achieved high and similar levels of Treg purity (VP 95.9 ± 2.3, 25MP 95.1 ± 3.7 vs. DP 94.5 ± 3.1%, mean ± SD, *p* = 0.57, Figures 1B and 1C). Purity for Tregs in the enriched cell population was higher after 25MP compared to VP (58.8 ± 12.0 vs. 25.4 ± 13.0%, *p* < 0.001, Table 1).

**Table 1:** Regulatory T cell sorting parameters—comparing CD4+CD25+ VACS enrichment then VACS purity sort, CD25+ IMS enrichment then VACS purity sort, or direct VACS purity sort

Parameter	CD4+CD25+ VACS enrichment then VACS purity sort (VP)	CD25+ IMS enrichment then VACS purity sort (25MP)	Direct VACS purity sort (DP)
Treg purity in final product, %	95.9 ± 2.3	95.2 ± 3.7	94.5 ± 3.1
Number of Tregs × 10^6^ in final product per hour	0.55 ± 0.27	1.90 ± 1.87	0.29 ± 0.17
Number of PBMCs enriched and sorted × 10^6^ per hour	106.9 ± 24.7	462.5 ± 235.1	30.3 ± 1.3
Tregs remaining in negative fraction after enrichment, %	55.8 ± 13.8	59.3 ± 21.3	NA

Values are expressed as mean ± SD.

VACS, vortex-actuated cell sorting; IMS, immunomagnetic separation; Treg, regulatory T cells; PBMCs, peripheral blood mononuclear cells; NA, not applicable.

VP obtained more Tregs over time compared to DP (0.55 ± 0.27 vs. 0.29 ± 0.17 × 10^6^ Tregs per hour, *p* = 0.01) but fewer than 25MP (0.55 ± 0.27 vs. 2.06 ± 1.83 × 10^6^ Tregs per hour, *p* = 0.01). Yield was higher in VP compared to 25MP (13.7 ± 4.0 vs. 9.6 ± 4.3%, *p* = 0.03) but lower than DP (13.7 ± 4.0 vs. 25.6 ± 9.8%, *p* = 0.006, Figure 1D).

The total time required for both enrichment and purity sorts per 10 × 10^6^ PMBCs in the starting material was lower in VP compared to DP (5.9 ± 1.3 vs. 19.8 ± 0.8 min per 10 × 10^6^ PMBCs, *p* < 0.001, Figure 1E) but higher than 25MP (5.9 ± 1.3 vs. 1.4 ± 0.5 min per 10 × 10^6^ PMBCs, *p* < 0.001).

IMS and VACS enrichment or sorting divide the initial sample into a positive fraction containing the cells of interest and a negative fraction containing cells from the initial sample that are partially depleted of the cells of interest. The degree of depletion for Tregs, defined as the percentage reduction of Tregs remaining in the negative fraction compared to that in the initial sample, as an indication of the proportion of Tregs that has been left behind after enrichment, was not different between CD4+CD25+ VACS enrichment and CD25+ IMS enrichment (55.8 ± 13.8 vs. 59.3 ± 21.3%, *p* = 0.69).

### CD4+CD25+ VACS enrichment improved early expansion and produced Tregs with similar phenotypic and functional characteristics compared to CD25+ IMS enrichment

**Figure 2:**
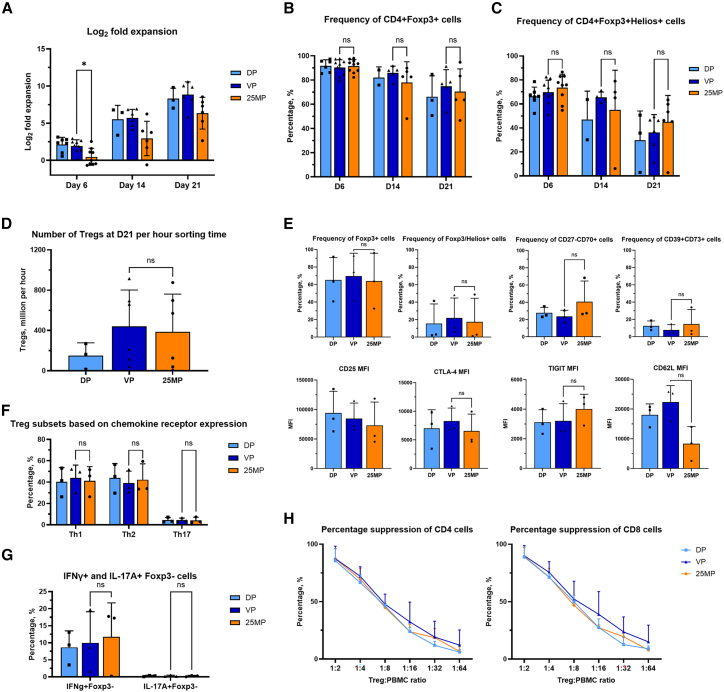
Phenotypic and functional characteristics of regulatory T cells with *ex vivo* expansion on days 6, 14, and 21 after CD4+CD25+ vortex-actuated cell sorting enrichment followed by vortex-actuated cell sorting purity sort, CD25+ immunomagnetic separation enrichment followed by vortex-actuated cell sorting purity sort, or direct VACS purity sort (A) Log_2_ fold expansion normalized to Treg cell counts on day 0 over time, *n* = 9 donors. (B) Frequency of CD4+Foxp3+ cells over time, *n* = 9 donors. (C) Frequency of CD4+Foxp3+Helios+ double-positive cells over time, *n* = 9 donors. (D) Sorting time expressed as the number of *ex vivo* expanded Tregs in the final product obtained at day 21 per hour of initial sorting time, *n* = 6 donors. (E) Flow cytometric analysis of phenotypic and functional markers of *ex vivo* expanded Tregs on day 21, *n* = 3 donors. (F) Frequency of Tregs subsets based on chemokine receptor expression (CXCR3+CCR6-, CXCR3-CCR4+CCR6-, and CXCR3-CCR4+CCR6+ were classified as Th1-, Th2-, and Th17-like Tregs respectively), *n* = 3 donors. (G) Frequency of interferon-gamma and IL-17A producing Foxp3-cell on day 21, *n* = 3 donors. (H) Suppression function of day 21 Tregs, expressed as percentage suppression of division index, *n* = 3 donors. Direct VACS purity sort (DP), CD4+CD25+ vortex-actuated cell sorting enrichment followed by VACS purity sort (VP), or CD25+ immunomagnetic separation (IMS) enrichment followed by VACS purity sort (25MP). Each symbol represent individual data points; bars indicate the mean ± SD. For (A)–€ paired *t* tests were performed. “∗” denotes a significance of *p* < 0.05.

To compare the phenotypic and functional characteristics of the Tregs produced by the different sorting modalities, we expanded the sorted Tregs *ex vivo* for up to 21 days. Tregs sorted by VP expanded more than 25MP after 6 days (1.91 ± 0.86 vs. 0.47 ± 1.14 log_2_ fold expansion, *p* = 0.003) and 14 days (5.71 ± 1.14 vs. 2.94 ± 2.32 log_2_ fold expansion, *p* = 0.03). At day 21, *ex vivo* expansion of VP did not differ significantly from that of 25MP, although it trended toward significance (5.71 ± 1.14 vs. 2.94 ± 2.32 log_2_ fold expansion, *p* = 0.054, Figure 2A). The ratio of log_2_ fold expansion for VP to 25MP decreased from 4.10 on day 6 to 1.94 on day 14 to 1.39 on day 21.

The frequency of CD4+Foxp3+ and CD4+Foxp3+Helios+ cells reduced over time but did not differ between VP and 25MP (Figures 2B and 2C). The number of Tregs in the final product on day 21 per hour of initial total enrichment and sorting time did not differ between VP and 25MP (439.3 ± 361.7 vs. 385.7 ± 374.4 × 10^6^ cells per hour, *p* = 0.73, Figure 2D). There were no significant differences in log_2_ fold expansion of non-CD4+Foxp3+ populations between groups at day 14 (DP 5.8 ± 0.1, VP 4.5 ± 1.7, 25MP 3.4 ± 1.9) and day 21 (DP 8.5 ± 0.7, VP 8.7 ± 0.5, 25MP 7.2 ± 2.4, Figures S1 and S2).

Phenotypic markers, cytokine production, chemokine receptor expression, and suppression function did not differ significantly after expansion for 21 days (Figures 2E–2H).

Based on the mean processing time, yield and expansion fold for VP and assuming 500 × 10^6^ PBMCs in the starting material, the estimated times for enrichment and purity sort are 3.7 and 1.3 h, respectively, yielding 2.5 × 10^6^ Tregs (Table S2). The numbers of Tregs after 6, 14, and 21 days of expansion were estimated to be 9.3 × 10^6^, 130 × 10^6^, and 1,114 × 10^6^ cells, respectively, which provides adequate Treg numbers based on dosing regimens of current clinical trials.

### Tregs sorted by CD4+CD25+ VACS enrichment showed increased early viability and responsiveness to IL-2 stimulation

**Figure 3:**
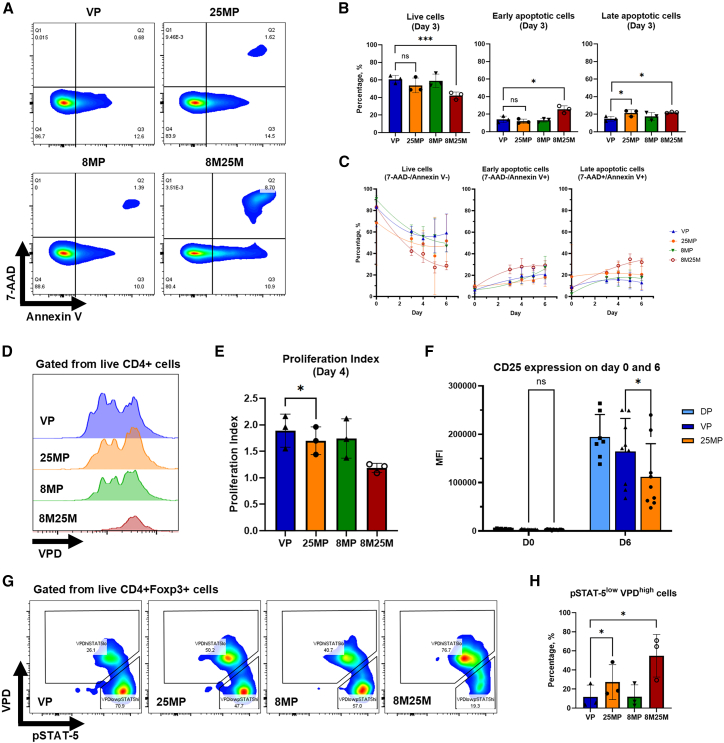
Assessment of viability, proliferation, and IL-2 responsiveness of human regulatory T cells sorted by CD4+CD25+ vortex-actuated cell sorting enrichment followed by vortex-actuated cell sorting purity sort, CD25+ immunomagnetic separation enrichment followed by vortex-actuated cell sorting purity sort, or direct vortex-actuated cell sorting purity sort (A) Representative flow cytometry plots to evaluate cell viability based on 7-Aminoactinomycin D (7-AAD) and Annexin V staining—live (Annexin V-/7-AAD-), early apoptotic (Annexin V+/7-AAD-), and late apoptotic (Annexin V+/7-AAD+) cells. (B) Frequency of live, early apoptotic and late apoptotic cells on day 3 expansion, *n* = 3 donors. (C) Frequency of live, early apoptotic, and late apoptotic cells over time after sorting, *n* = 3 donors. (D) Representative proliferation plots of Tregs stained by violet proliferation dye (VPD) on day 4 of expansion. (E) Proliferation indices of Tregs on day 4 expansion, *n* = 3 donors. (F) CD25 expression on Tregs on day 0 and 6 expansion, *n* = 9 donors. (G) Representative flow cytometry plots showing presence of poorly proliferating and IL-2 hyporesponsive population (pSTAT-5^low^/VPD^high^) on day 6 expansion. (H) Frequencies of poorly proliferating and IL-2 hyporesponsive population (pSTAT-5^low^/VPD^high^) on day 6 expansion, *n* = 3 donors. Direct VACS purity sort (DP), CD4+CD25+ vortex-actuated cell sorting enrichment followed by VACS purity sort (VP), CD25+ immunomagnetic separation (IMS) enrichment followed by VACS purity sort (25MP), CD8+ IMS negative enrichment followed by VACS purity sort (8MP), and CD8+ IMS negative enrichment followed by CD25+ IMS positive enrichment (8M25M). Each symbol represent individual data points; bars indicate the mean ± SD. For (B), (E), (F), and (H) paired *t* tests were performed. “∗” denotes a significance of *p* < 0.05.

To explore the possible mechanisms that may account for the differences in expansion capacity between Tregs sorted by VP and 25MP, we evaluated the viability and proliferation of Tregs sorted by VP and 25MP 6 days after sorting (Figure 3A). For comparison, we sorted Tregs from PBMCs using CD8 microbeads for negative IMS selection, followed by VACS purity sort (8MP) or CD25 microbeads positive selection (8M25M). The frequency of late apoptotic cells on day 3 was higher for 25MP than VP (21.6 ± 3.6 vs. 14.8 ± 2.7%, *p* = 0.03) and for 8M25M than VP (22.1 ± 0.7 vs. 14.8 ± 2.7%, *p* = 0.03, Figure 3B). The frequency of early apoptotic cells on day 3 was also higher for 8M25M compared to VP (25.5 ± 4.1 vs. 14.1 ± 3.7%, *p* = 0.04), while live cells were lower (42.2 ± 4.1 vs. 60.8 ± 4.4%, *p* < 0.001). When the data points were fitted over a quadratic curve, the frequency of live Tregs started to improve at an earlier time for VP compared to 25MP (4.5 vs. 5.1 days, Figure 3C). At day 4, the proliferation index of Tregs sorted by VP was higher than 25MP (1.89 ± 0.31 vs. 1.70 ± 0.26, *p* = 0.048), while division indices did no differ (0.91 ± 0.45 vs. 0.85 ± 0.46, *p* = 0.76, Figures 3D and 3E).

To explore the impact of CD25+ IMS on CD25 expression, we trended CD25 expression over time. CD25 expression, measured by median fluorescent intensity (MFI), did not differ between VP and 25MP immediately after sorting (3,095 ± 743 vs. 2,733 ± 927, *p* = 0.22) but was higher in VP on day 6 of expansion (164,713 ± 68,282 vs. 112,048 ± 68,433, *p* = 0.02, Figure 3F). To assess IL-2 responsiveness, we performed phospho-flow staining on violet proliferation dye (VPD)-stained Tregs that were expanded for 6 days, then rested for 2 days without IL-2. Staining for phosphorylated STAT-5 (pSTAT-5) after IL-2 stimulation revealed 2 distinct populations in the Tregs sorted by each modality (Figure 3G)—an IL-2 responsive, proliferating (pSTAT-5^high^/VPD^low^) population and an IL-2 hyporesponsive, poorly proliferating (pSTAT-5^low^/VPD^high^) population. The frequency of pSTAT-5^low^/VPD^high^ cells was lower in VP than in 25MP (11.8 ± 12.4 vs. 27.3 ± 18.3%, *p* = 0.045, Figure 3H).

## Discussion

Our study demonstrated that VACS sorting after CD4+CD25+ VACS enrichment can reduce sorting time compared to direct VACS sorting and produce Tregs of high purity, comparable to 25MP and DP. While VP was slower than 25MP, it yielded more Tregs and produced Tregs that expanded better initially, with comparable phenotypic and functional characteristics after expansion. Further investigation revealed that Tregs sorted by VP may potentially have improved viability, faster recovery, increased proliferation, and enhanced IL-2 responsiveness compared to those sorted by 25MP. Early fitness following VACS may therefore reduce culture failure and is likely to reduce time to dose.

Pre-enrichment before sorting for rare cell populations has been recommended to reduce sorting time.^30^ While other pre-enrichment methods have been described, CD25+ IMS is one of the most commonly used methods for sorting human Tregs.^22^^,^^26^^,^^27^ Consistent with our findings, previous studies have demonstrated that Tregs sorted using IMS may have reduced expansion and cell viability.^14^^,^^16^ Gedaly et al. have shown that the superparamagnetic iron oxide nanoparticles used to label cells for IMS were internalized by Tregs and may contribute to increased mitochondrial oxidative stress.^16^ We observed decreased CD25 expression and decreased IL-2 responsiveness in Tregs sorted by 25MP, though it is unclear whether these changes are caused directly by CD25 downregulation or result from reduced cell viability and metabolic derangements. These adverse effects may be mitigated by utilizing IMS negative selection to remove unwanted cells and select for “untouched” target cells. However, such as approach will require a higher number of specific antibodies and increase the cost of sorting.^12^ Importantly, the differences between cells sorted by different techniques observed in our study and others appear to be transient and have limited effect on the quantity and function of Tregs after a period of expansion.^15^^,^^16^^,^^31^ Results from other studies comparing IMS vs. FACS for T cells and other cell types have been limited and mixed, suggesting that these differences may be specific to certain sorting conditions or cell types.^12^^,^^13^^,^^23^^,^^32^

IMS is able to process large numbers of cells in bulk and therefore can pre-enrich cells at a faster rate compared to VACS, especially when processing cells at larger scale, such as in a GMP setting. Cell loss and decreased yield, which have been described in a previous study utilizing sequential IMS followed by FACS, may be mitigated by VP.^15^ Sorter-induced cell stress caused by FACS has also been described in numerous studies and may be contributed to by factors such as the shear force experienced by the cells as they are pushed through the nozzle at high pressures and exposure to undesirable conditions in the sheath fluid.^15^^,^^33^ However, microfluidics-based sorters may be gentler and therefore allow cells to tolerate sequential enrichment followed by sorting.^23^^,^^30^^,^^34^^,^^35^^,^^36^ While our data are promising, it is important to note that we used leukocyte cones from healthy donors rather than patient material, and expansion was assessed *ex vivo* rather than *in vivo*.

In conclusion, CD4+CD25+ VACS enrichment may be a reasonable alternative to direct VACS or CD25+ IMS enrichment prior to VACS for human Tregs. Although differences at day 21 converged across workflows, suggesting comparable long-term functionality, the early fitness advantages with VP are relevant to batch reliability and time to dose in manufacturing. By combining pre-enrichment and purity sorting on a single closed microfluidic platform, VP shortens sorting and improves early cell fitness without compromising the day 21 product—a balance that is well suited for clinical manufacturing.

## Materials and methods

### PBMC isolation, Tregs sorting, and expansion

Donor leukocyte cones were generated from blood collected from anonymous, healthy donors the day before each experiment by NHS Blood and Transplant (NHSBT), United Kingdom, and used with informed, written pre-consent and ethical approval from the Oxfordshire Research Ethics Committee B (#07/H0605/130). All reagents, antibodies and software used in this study are listed in the Key Resources Table (Table 2).

**Table 2:** Key resources table

REAGENT or RESOURCE	SOURCE	IDENTIFIER
**Antibodies**		

Mouse monoclonal anti-human CD4 (clone OKT4)	BioLegend	CAT: 317407, RRID: AB_571950
Mouse monoclonal anti-CD25 (clone BC96)	BioLegend	CAT: 302605, RRID: AB_314275
Mouse monoclonal anti-human CD127 (clone A019D5)	BioLegend	CAT: 351315, RRID: AB_10900814
Mouse monoclonal anti-human CD25 (clone M-A251)	BD	CAT: 557741, RRID: AB_396847
Mouse monoclonal anti-human CD62L (clone DREG-56)	BioLegend	CAT: 304859, RRID: AB_2860806
Mouse monoclonal anti-human TIGIT (clone A15153)	BioLegend	CAT: 372703, RRID: AB_2632729
Mouse monoclonal anti-human CD27 (clone O323)	Invitrogen	CAT: 48-0279-42, RRID: AB_10852844
Mouse monoclonal anti-human human CD39 (clone A1)	BioLegend	CAT: 328205, RRID: AB_940423
Mouse monoclonal anti-human CD70 (clone 113-16)	BioLegend	CAT: 355104, RRID: AB_2561431
Mouse monoclonal anti-human CD73 (clone AD2)	BioLegend	CAT: 344009, RRID: AB_2561541
Rat monoclonal anti-human Foxp3 (clone PCH101)	eBioscience	CAT: 12-4776-42, RRID: AB_1518782
Mouse monoclonal anti-human Foxp3 (clone 206D)	BioLegend	CAT: 320112, RRID: AB_430883
Rat monoclonal anti-human Foxp3 (clone PCH101)	Invitrogen	CAT: 48-4776-42, RRID: AB_1834364
Armenian Hamster monoclonal anti-human Helios (clone 22F6)	BioLegend	CAT: 137218, RRID: AB_10660750
Mouse monoclonal anti-human CTLA4 (clone BNI3)	BioLegend	CAT: 369605, RRID: AB_2616790
Mouse monoclonal anti-human CXCR3 (clone G025H)	BioLegend	CAT: 353710, RRID: AB_10962442
Mouse monoclonal anti-human CCR4 (clone L291H4)	BioLegend	CAT: 359410, RRID: AB_2562431
Mouse monoclonal anti-human CCR6 (clone G034E3)	BioLegend	CAT: 353436, AB_2629608
Mouse monoclonal anti-human CRTH2 (clone BM16)	BioLegend	CAT: 350104, RRID: AB_10642025
Mouse monoclonal anti-human CCR7 (clone G043H7)	BioLegend	CAT: 353208, RRID: AB_11203894
Rat monoclonal anti-human GATA-3 (clone TWAJ)	Invitrogen	CAT: 50-9966-42, RRID: AB_10596663
Rat monoclonal anti-human RORγt (clone Q21-559)	BD	CAT: 563081, RRID: AB_2686896
Mouse monoclonal anti-human IFN-γ (clone 4S.B3)	BioLegend	CAT: 502538, RRID: AB_2563608
Rat monoclonal anti-human IL4 (MP4-25D2)	BioLegend	CAT: 500817, RRID: AB_493324
Mouse monoclonal anti-human IL-17A (BL168)	BioLegend	CAT: 512338, RRID: AB_2566765
Rat monoclonal anti-human IL-10 (JES3-9D7)	BioLegend	CAT: 501420, RRID: AB_2125385
Mouse monoclonal anti-human CD3 (clone OKT3)	Invitrogen	CAT: 47-0037-42, RRID: AB_2573936
Mouse monoclonal anti-human CD4 (clone RPA-T4)	Invitrogen	CAT: 61-0049-42, RRID: AB_2574522
Mouse monoclonal anti-human CD8 (clone HIT8a)	BioLegend	CAT: 300906, RRID: AB_314110
Mouse monoclonal anti-human STAT5 phospho (Tyr694) (clone A17016B.Rec)	BioLegend	CAT: 936905, RRID: AB_2892500

**Biological samples**		

Healthy donor leukocyte blood cones	NHS Blood and Transplant	N/A

**Chemicals, peptides, and recombinant proteins**		

Lymphocyte Separation Medium 1077	PromoCell	CAT: C-44010
7-Aminoactinomycin D viability staining solution	Invitrogen	CAT: 00-6993-50
Lysing buffer	BD	CAT: 555899
Poloxamer 188 non-ionic surfactant	Thermo Fisher Scientific	CAT: 24040032
Benzonase nuclease	Merck Life Science	CAT: E1014-5KU
Magnesium chloride	Merck Life Science	CAT: M8266-100g
Roswell Park Memorial Institute (RPMI) medium	Life Technologies	CAT: 21870076
L-Glutamine	Life Technologies	CAT: 25030024
Penicillin/Streptomycin	Life Technologies	CAT: 15140122
Rapamycin	Miltenyi Biotec	CAT: 170-076-308
Aldesleukin (recombinant human IL-2, Proleukin)	Clinigen Healthcare	CAS: 110942-02-4
Pooled AB-negative male human serum	Merck Life Science	CAT: H5667-100ML
Foetal bovine serum	Gibco	CAT: A5209402
Zombie NIR fixable viability kit	BioLegend	CAT: 423105
Foxp3/transcription Factor Staining Buffer Set	eBioscience	CAT: 00-5523-00
Normal mouse serum	eBioscience	CAT: 24-5544-94
Normal rat serum	eBioscience	CAT: 24-5555-93
Phorbol myristate acetate (PMA)/ionomycin (Cell Stimulation Cocktail)	eBioscience	CAT: 00-4970-93
Monensin	Life Technologies	CAT: 00-4505-51
Violet proliferation dye 450	BD	CAT: 562158
Annexin V	BioLegend	CAT: 640949
Annexin V binding buffer	BioLegend	CAT: 422201
Methanol, ≥99%	Merck Life Science	CAT: 34860-2.5L-R
Formaldehyde 36% (39% W/V)	VWR International	CAT: 20910.328

**Software and algorithms**		

FlowJo version 10	BD	RRID: SCR_008520
Prism version 10	GraphPad	RRID: SCR_002798

**Other**		

LS Columns	Miltenyi Biotec	130-042-401
CD25 microbeads II	Miltenyi Biotec	CAT: 130-092-983
CD8 microbeads	Miltenyi Biotec	CAT: 130-045-201
Human T-Activator CD3/CD28 stimulation Dynabeads	Life Technologies	CAT: 11132D

PBMCs were isolated using the standard gradient centrifugation approach with lymphocyte separation medium 1077 (PromoCell, Germany) and immediately used for experiments. PBMCs from the same donor leukocyte cone were sorted using different techniques to generate matched samples for comparison.

For VACS enrichment, PBMCs were stained with anti-CD4 FITC (OKT4), anti-CD25 PE (BC96), anti-CD127 APC (A019D5, all BioLegend), and 7-aminoactinomycin D (7-AAD) staining solution (Invitrogen), diluted to 8 × 10^6^ cells/ml in PBS with 0.1% Poloxamer 188 non-ionic surfactant (Thermo Fisher Scientific), 0.5 mM MgCl2, and benzonase nuclease at 24 IU/ml (Merck Life Science), then enriched for CD4+CD25+ cells using the enrichment mode of the Highway1 cell sorter (Cellular Highways, United Kingdom).

CD25+ positive and CD8+ negative IMS enrichment were performed using CD25 microbeads II and CD8 microbeads, respectively, with LS columns according to the manufacturer’s instructions (Miltenyi Biotec, Germany). The eluted magnetically bound cells from CD25 positive enrichment and the cells in the flow-through for CD8+ negative enrichment were used for further sorting. IMS-enriched cells were then stained with the same antibodies and buffers as used for VACS enrichment (as stated above) and diluted to 2 × 10^6^ cells/ml.

CD4+CD25^high^CD127^low^ Tregs were finally sorted immediately either from VACS-enriched (VP), CD25+ IMS positively enriched (25MP), CD8+ IMS negatively enriched (8MP) cells, or directly from stained PBMCs (DP) at a maximum concentration of 2 × 10^6^ cells/ml using the purity mode of the Highway1. All VACS enrichment and sorting procedures, including cell concentrations and buffers used, were performed according to the manufacturer’s instructions. As an alternative, PBMCs were enriched for Tregs by sequential CD8+ IMS negative selection followed by CD25+ IMS positive selection (8M25M). No additional purity sort after expansion was performed.

Sort purities, as defined as the percentage of live CD4+CD25^high^CD127^low^ cells of total live cells, of the enriched and final cell populations were assessed using FACSCanto II (BD). Yield was defined as the percentage of Tregs recovered in the final product relative to the number of Tregs in the initial PBMC starting material, i.e., number of live CD4+CD25^high^CD127^low^ cells in the final product/number of live CD4+CD25^high^CD127^low^ cells in the initial PBMC sample.

IMS and VACS enrichment or sorting divides the initial sample into a positive fraction containing the cells of interest and a negative fraction containing cells from the initial sample that are partially depleted of the cells of interest. The degree of depletion for Tregs was defined as the percentage reduction of Tregs remaining in the negative fraction compared to that in the initial sample, i.e., (percentage of Tregs in initial sample – percentage of Tregs in negative fraction)/percentage of Tregs in initial sample, as an indication of the proportion of Tregs that has been left behind after enrichment. All sorts were performed by the same operator using a fixed gating template and matched flow rates.

Sorted Tregs were seeded on 96-well plates (Scientific Laboratory Supplies) at 0.1 × 10^6^ cells with 0.3 × 10^6^ Human T-Activator CD3/CD28 stimulation Dynabeads (Life Technologies) per well in 200 uL of Roswell Park Memorial Institute (RPMI) medium (Life Technologies) supplemented with 2 mM glutamine, 100 U/ml penicillin, 0.1 mg/ml streptomycin (Merck Life Science), 100 nM rapamycin (Miltenyi Biotec, Germany), 1000 IU/ml IL-2 (Proleukin, Clinigen Healthcare), and 10% pooled AB-negative male human serum (Merck Life Science). Fresh media was replenished every 2–3 days. Cells were re-stimulated every 7 days with CD3/CD28 stimulation Dynabeads at 1:1 cell-to-bead ratio.

### Phenotypic assessment of expanded Tregs

Cells were stained for phenotypic and functional markers of interest at specified timepoints before and after cell sorting and expansion. Surface markers CD25+ (M-A251, BD), CD62L (DREG-56, BioLegend), TIGIT (A15153G, BioLegend), CD27 (O323, Invitrogen), CD70 (113-16, BioLegend), CD39 (A1, BioLegend), and CD73 (AD2, BioLegend) were stained for 15 min at 4°C. Intracellular markers Foxp3 (PCH101, eBioscience; 206D, BioLegend, or PCH101, Invitrogen), Helios (22F6, BioLegend), and CTLA-4 (BNI3, BioLegend) were stained for 30 min in 4°C after permeabilization/fixation (Foxp3/Transcription Factor Staining Buffer Set, eBioscience). For chemokine receptors CXCR3 (G025H), CCR4 (L291H4), CCR6 (G034E3), CRTH2 (BM16), and CCR7 (G043H7, all BioLegend), cells were stained at 37°C for 30 min. Tregs that were CXCR3+CCR6-, CXCR3-CCR4+CCR6-, and CXCR3-CCR4+CCR6+ were classified as Th1-, Th2-, and Th17-like Tregs, respectively. For intracellular transcription factors GATA-3 (TWAJ, Invitrogen), RORγt (Q21-559, BD), Foxp3 (PCH101, Invitrogen), and cytokines IFN-γ (4S.B3), IL4 (MP4-25D2), IL-17A (BL168), and IL-10 (JES3-9D7, all BioLegend), cells were stained after stimulation with phorbol myristate acetate (PMA)/ionomycin (Cell Stimulation Cocktail, eBioscience) for 2.5 h and incubated with monensin (Life Technologies) for 2.5 h. Samples were acquired on the Attune NxT flow cytometer (Thermo Fisher Scientific).

### *In vitro* Treg suppression assay

Cryopreserved autologous PBMCs (Tresp) were thawed, stained with VPD 450 (BD), and co-cultured at 0.5 × 10^5^ cells per well with 0.1 × 10^5^ human T-Activator CD3/CD28 stimulation Dynabeads (Life Technologies), with expanded Tregs at different ratios (Tregs:Tresp 1:2 to 1:128) in RPMI supplemented with 2 nM glutamine, 100 U/ml penicillin, 0.1 mg/ml streptomycin (Merck Life Science), and 10% fetal calf serum (Gibco) in 96-well plates. Tresp with stimulation beads without Tregs served as positive controls, while Tresp alone conditions served as negative controls. After approximately 80 h, cells were harvested and stained with CD3 (OKT3, Invitrogen), CD4 (RPA-T4, Invitrogen), CD8 (HIT8a, BioLegend), and 7-AAD (Invitrogen) and acquired on the BD FACSCanto II (BD Biosciences). Suppression function was presented as the percentage suppression of division index (DI)—i.e., (DI of Tresp with stimulation beads without Tregs—DI of Treg-treated Tresp)/(DI of Tresp with stimulation beads without Tregs).

### Cell proliferation and viability assay

Tregs isolated from PBMCs were stained with VPD 450 (BD Biosciences) and expanded as described above. Tregs were taken on D0, 3 to 6, washed twice with PBS, stained with anti-CD4 (OKT4), anti-CD25 (BC96), and anti-CD127 (A019D5), resuspended in Annexin V binding buffer (BioLegend), and stained with Annexin V (all BioLegend) and 7-AAD (Invitrogen) before acquisition by FACSCanto II (BD Biosciences). The frequencies of live, early apoptotic, and late apoptotic cells were plotted daily from day 0 to day 6 of expansion. Polynomial quadratic curves (Y=B_0_ + B_1_∗X + B_2_∗X^2^) were fitted over data points with the *y* axis as the percentage of live or apoptotic cells using GraphPad Prism version 10.

### Detection of phosphorylated STAT5 after IL-2 stimulation by phospho-flow

Tregs were separated from CD3/28 stimulation beads on day 6 of expansion and rested for 48 h in complete medium without IL-2. Cells were then stained with Zombie NIR (BioLegend) and then stimulated with IL-2 at 1000 IU/ml. Cells were fixed directly at 0 and 30 min with 2% formaldehyde at 37°C and incubated for 10 min. Samples were then permeabilized with ice-cold 90% methanol and incubated for 30 min at 4°C. Cells were subsequently stained with anti-CD4 (RPA-T4, BioLegend), anti-Foxp3 (PCH101, Invitrogen), and anti-STAT5 Phospho (Tyr694) (A17016B.Rec, BioLegend) before acquisition by BD FACSCanto II (BD Biosciences).

### Data and statistical analysis

Flow cytometry data was analyzed on FlowJo software version 10 (BD). Statistical analyses were performed using GraphPad Prism version 10. Paired *t* tests and repeated measures one-way ANOVA were used to compare data derived from the same blood donor for continuous data with 2 or more than 2 groups, respectively. A *p*-value of less than 0.05 was considered statistically significant.

## Data availability

The data supporting this study are available from the corresponding author upon reasonable request.

## Acknowledgments

Experimental work was funded by the ReSHAPE project under the 10.13039/501100007601European Union’s Horizon 2020 research and innovation program (grant agreement no. 825392) and supported by the Medical Research Council, United Kingdom (grant number: MR/N027930/1) and the Chinese Academy of Medical Sciences (CAMS) Innovation Fund for Medical Science (CIFMS), China (grant number: 2024-I2M-2-001-1).

Q.Y.H. is supported by funding from the Singapore General Hospital Research Training Fellowship and the National Medical Research Council Singapore Research Training Fellowship.

## Author contributions

Conceptualization, Q.Y.H. and FI; data curation, Q.Y.H.; formal analysis, Q.Y.H.; funding acquisition, J.H. and F.I.; investigation, Q.Y.H.; methodology, all; project administration, Q.Y.H.; resources, H.H., J.H., and F.I.; software, Q.Y.H.; supervision, J.H. and F.I.; validation, H.H., J.H., and F.I.; visualization, Q.Y.H.; writing – original draft, Q.Y.H.; writing – review & editing, all.

## Declaration of interests

Cellular Highways (TTP plc, United Kingdom) provided access to the Highway1 cell sorter together with the relevant consumables but had no role in the design of the study, data collection, analysis, or interpretation of the results.

## Supplemental information

Document S1. Figures S1, S2, Tables S1, and S2

Document S2. Article plus supplemental information
