# Supplementary material for: Vortex-actuated pre-enrichment accelerates human regulatory T cell sorting and improves early fitness
Source: Mol Ther Adv. 2026 Jan 9;34(1):201667. doi: 10.1016/j.omta.2026.201667 (PMC13148893; doi:10.1016/j.omta.2026.201667)
Supplement: Document S1. Figures S1, S2, Tables S1, and S2 [file mmc1.pdf]

**OMTA, Volume *34***

## Supplemental information

# Vortex-actuated pre-enrichment accelerates human regulatory T cell sorting and improves early fitness

**Quan Yao Ho, Hisashi Hashimoto, Joanna Hester, and Fadi Issa**

**Table S1.** Breakdown of enrichment and sorting parameters based on number of peripheral blood mononuclear cells in starting material

| | DP | VP | | | 25MP | | |
|---|---|---|---|---|---|---|---|
| | N=9 | N=2 | N=7 | N=3 | N=2 | N=7 | N=3 |
| Starting PBMCs, x$10^6$ cells | 40 | 80 | 160 | 200 | 80 | 160 | 400 |
| Enrichment time, minutes | NA | 40.5 ±27.6 | 68.6 ±23.6 | 83.5 ±4.5 | 9.5 ±0.7 | 17.3 ±2.0 | 12.7 ±0.6 |
| PBMCs after sorting enrichment, x$10^6$ cells | NA | 3.1 ±1.1 | 9.8 ±10.6 | 12.2 ±2.6 | 2.0 ±1.6 | 3.0 ±1.3 | 9.9 ±3.0 |
| Sorting time, minutes | 79.3 ±3.2 | 10.5 ±3.5 | 27.1 ±13.8 | 23.1 ±1.4 | 7.5 ±4.9 | 6.7 ±2.1 | 18.1 ±6.3 |
| Total enrichment and sorting time, minutes | 79.3 ±3.2 | 51 ±31.1 | 95.7 ±11.6 | 106.5 ±5.9 | 17.0 ±5.7 | 24.0 ±2.9 | 30.8 ±6.6 |
| Tregs obtained after enrichment/sorting, x$10^6$ cells | 0.4 ±0.3 | 0.3 ±0.2 | 0.7 ±0.5 | 1.7 ±0.1 | 0.3 ±0.1 | 0.5 ±0.3 | 2.1 ±0.6 |

Data presented as mean±SD. DP, direct purity sort; VP, CD4+CD25+ vortex-actuated cell sorting (VACS) enrichment followed by VACS purity sort; 25MP, CD25 immunomagnetic separation enrichment followed by VACS purity sort; PBMCs, peripheral blood mononuclear cells; Tregs regulatory T cells; NA, not applicable

**Table S2.** Estimated time needed for enrichment and purity sort and numbers of regulatory T cells (Tregs) obtained after enrichment and purity sort and expansion, based on 500x$10^6$ peripheral blood mononuclear cells (PBMCs) in starting material using mean enrichment time, purity sorting time, yield and $\log_2$-fold expansion of each modality

| | DP | VP | 25MP |
|---|---|---|---|
| Number of PBMCs in starting material, x$10^6$ cells | 500 | | |
| Time for enrichment, hours | NA | 3.7 | 1.0 |
| Time for purity sort, hours | 16.5 | 1.3 | 0.4 |
| Total time for enrichment and purity sort, time | 16.5 | 5.0 | 1.4 |
| Number of Tregs obtained after enrichment/purity sort, x$10^6$ cells | 4.6 | 2.5 | 1.7 |
| Number of Tregs obtained after 6 days expansion, x$10^6$ cells | 19.7 | 9.3 | 2.4 |
| Number of Tregs obtained after 14 days expansion, x$10^6$ cells | 208 | 130 | 12.7 |
| Number of Tregs obtained after 21 days expansion, x$10^6$ cells | 1450 | 1114 | 138 |

DP, direct purity sort; VP, CD4+CD25+ vortex-actuated cell sorting (VACS) enrichment followed by VACS purity sort; 25MP, CD25 immunomagnetic separation enrichment followed by VACS purity sort; PBMCs, peripheral blood mononuclear cells; Tregs regulatory T cells

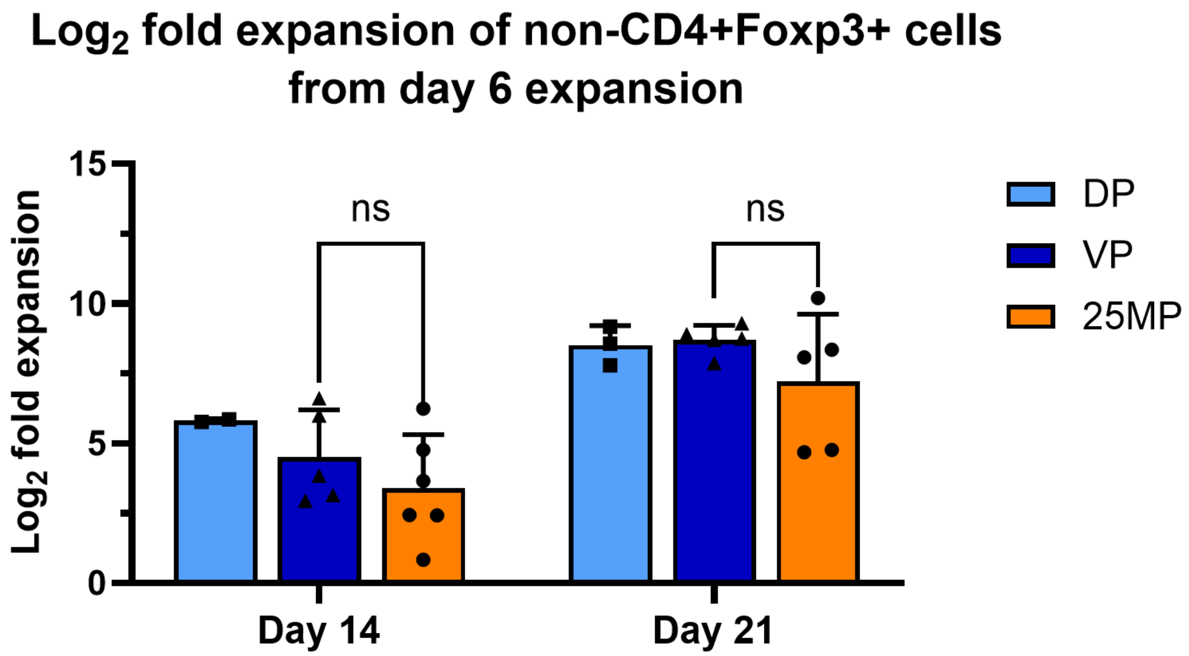


**Figure S1. Log2 fold expansion of non-CD4+Foxp3+ cells on day 14 and day 21 from day 6 ex vivo expansion.**

Direct VACS purity sort (DP), CD4+CD25+ vortex-actuated cell sorting enrichment followed by VACS purity sort (VP) or CD25+ immunomagnetic separation (IMS) enrichment followed by VACS purity sort (25MP). Paired t-tests were performed

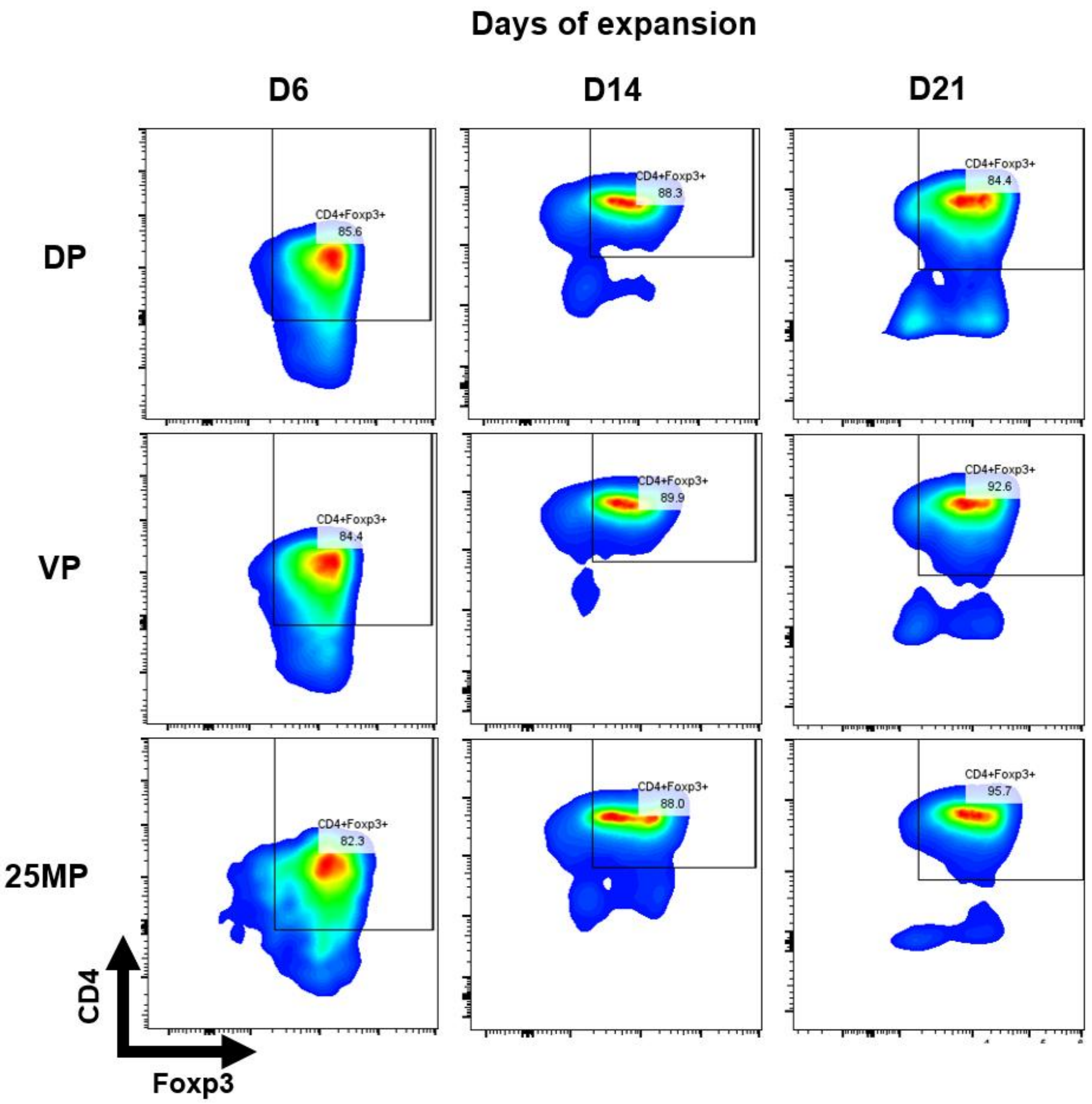


**Figure S2. Representative flow cytometric plots comparing CD4+Foxp3+ populations with ex vivo expansion on days 6, 14 and 21.**

Direct VACS purity sort (DP), CD4+CD25+ vortex-actuated cell sorting enrichment followed by VACS purity sort (VP) or CD25+ immunomagnetic separation (IMS) enrichment followed by VACS purity sort (25MP). FoxP3 gating was determined using an FMO control.
